# Controlling of Pneumatic Muscle Actuator Systems by Parallel Structure of Neural Network and Proportional Controllers (PNNP)

**DOI:** 10.3389/frobt.2020.00115

**Published:** 2020-10-05

**Authors:** Alaa Al-Ibadi, Samia Nefti-Meziani, Steve Davis

**Affiliations:** ^1^School of Computing, Science and Engineering, University of Salford, Salford, United Kingdom; ^2^Computer Engineering Department, Engineering College, University of Basrah, Basrah, Iraq

**Keywords:** controller system, PMA, neural network, P controller, human-robot shared controller

## Abstract

This article proposed a novel controller structure to track the non-linear behavior of the pneumatic muscle actuator (PMA), such as the elongation for the extensor actuator and bending for the bending PMA. The proposed controller consists of a neural network (NN) controller laid in parallel with the proportional controller (P). The parallel neural network proportional (PNNP) controllers provide a high level of precision and fast-tracking control system. The PNNP has been applied to control the length of the single extensor PMA and the bending angle of the single self-bending contraction actuator (SBCA) at different load values. For further validation, the PNNP has been applied to control a human–robot shared control system. The results show the efficiency of the proposed controller structure.

## Introduction

Soft robotics represents a new generation of robotic research, which provides numerous advantages, such as being lightweight, safe for close contact with humans, and environmentally friendly, as well as having low cost in terms of materials, construction time, and power (Neppalli and Jones, 2007; Trivedi et al., 2008; Al-Ibadi et al., 2020). In addition to the general advantages of soft robotics, soft actuators, such as contraction and extension pneumatic muscle actuators (PMAs), have their benefits when compared with the traditional electrical and mechanical actuators. Moreover, there is a high ratio of force to the actuator weight, in most cases a 100 newtons for several 100 g (Tondu and Lopez, 2000; Al-Ibadi et al., 2017, 2018a; Yang et al., 2019), but on the other hand, due to the softness, low stiffness, and hysteresis, the PMA shows a high degree of non-linearity and adds more challenges to controlling such types of actuators (Wang et al., 2017; Giannaccini et al., 2018; Teramae et al., 2018).

The performances of soft robots provide infinite degrees-of-freedom (DoF) motions, such as elongation, contraction, bending, shrinkage, and rotation. Furthermore, different designs and actuation techniques give unique behaviors (Manti et al., 2016; Al-Ibadi et al., 2018b; George Thuruthel et al., 2018), and the value mechanism and the high rubber material non-linearity of the PMA make the control process difficult and rule out simple controllers. Therefore, to overcome these difficulties, the high robust control has to be considered (Tondu and Lopez, 2000; Leephakpreeda, 2011). Numerous types of control strategies were used to control the position and force of the PMA. Among them, a linear proportional–integral–derivative (PID) controller has been used in Andrikopoulos et al. (2011), Shen et al. (2015), and Chan et al. (2020). Four PID controllers have been used to control the orientation of the ankle rehabilitation robot of four PMAs, one controller for each actuator (Meng et al., 2017). Adaptive pole placement techniques for positioning PID controllers were applied in Bowler (1996). A sliding mode control was used in Cai and Yamaura (1997) and Carbonell et al. (2001), and a fuzzy sliding mode controller, which is trained by a neural network for single dimensional PMA, is used in Chiang and Chen (2017) and Chiang and Chen (2018). Fuzzy PID was used in Balasubramanian and Rattan (2003), and a fuzzy PD controller and an integration controller were used in Chan et al. (2003). Tracking control with hysteresis compensation was done by PID (Schreiber et al., 2011). A series combination of PID controllers and an artificial neural network (ANN)—non-linear PID—was used in Thanh and Ahn (2006) for physical rehabilitation by using multijoint actuate based on pneumatic muscles. Similar techniques have been used in Andrikopoulos et al. (2014).

This article aims to provide an efficient, simple structure, controller system to be used for various soft robotic systems. For that purpose, a parallel controller structure is proposed by using a neural network (NN) controller and a proportional (P) controller. This structure provides a fast and accurate response to track the soft pneumatic robot systems. The proposed controller has been used to control the position of single actuators, the bending angles of the self-bending contraction actuator (SBCA), and a human–robot shared control system to show the efficiency of the proposed controller for different robot behaviors and applications.

The order of this paper has been organized as follows: Section non-linear PID controller shows the idea of non-linear PID; Section other controller approaches describes several approaches to control the soft pneumatic systems. The proposed controller structure is presented in Section parallel neural network proportional controller together with its applications.

## Non-Linear PID Controller

**Figure 1:**
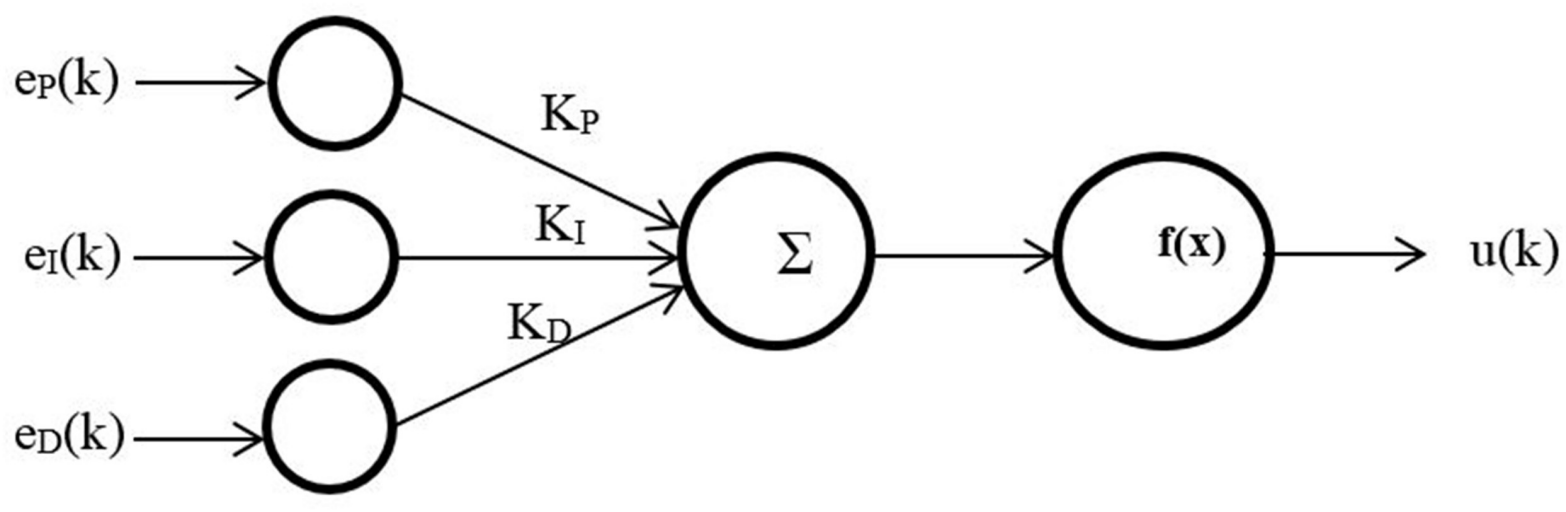
Proportional–integral–derivative (PID)-artificial neural network (ANN) control structure.

The PID controller has been one of the most important strategies used in industrial applications due to its simplicity and robustness. The need for variable efficient controller performance in operating conditions or parameters in the environment is often beyond the abilities of linear PID controllers (Su et al., 2005). Moreover, the high non-linearity of the PMAs makes the PID controller insufficient to solve this complex control problem. To improve the performance of linear PID to control the performances of PMA, numerous techniques have been utilized to enhance the performance and robustness of the PID controller by using the self-tuning method of general predictive control, fuzzy logic, and neural networks (Cervantes and Alvarez-Ramirez, 2001; Duan et al., 2004). Figure 1 shows the non-linear PID by connecting it serially to the ANN.

A multilayer ANN is used for three inputs, one neuron in one hidden layer and one output neuron, with

(1) $$\begin{array}{l} e_{P}\left(k\right)=\theta_{ref}\left(k\right)-\theta \left(k\right) \end{array}$$

(2) $$\begin{array}{l} e_{I}\left(k\right)=e_{P}\left(k\right)\Delta T \end{array}$$

(3) $$\begin{array}{l} e_{D}\left(k\right)=\frac{e_{P}\left(k\right)\left(1-z^{-1}\right)}{\Delta }T \end{array}$$

where θ_*ref*_ and θ are the setpoints and the actual output for each joint, respectively; Δ*T* is the sampling time; *z* is the Z-transform operator; *K*_*p*_, *K*_*I*_, and, *K*_*D*_ are PID constants that have to be modified to find the optimal value; *f* (*x*) is a sigmoid function; and *u*(*k*) is the controller output.

Other work was done in Anh (2010) using the same PID-ANN technique with added bias input to the hidden and output neurons.

## Other Controller Approaches

The inverse control strategy for PMA motion control was presented in Kang et al. (2013) and Kang et al. (2014). By using this idea, they were able to define an inverse kinematic (IK) model for control application. Furthermore, they assumed that the dynamics of the system could be ignored because the speed of these types of actuators is low. Meanwhile, Nakamura and Shinohara (2007) presented the controller system according to the mathematical model of PMA, which drives the inverse relationship between both the position and force of the PMA and the pressure input where *P* is the function of *L* and *F*.

**Figure 2:**
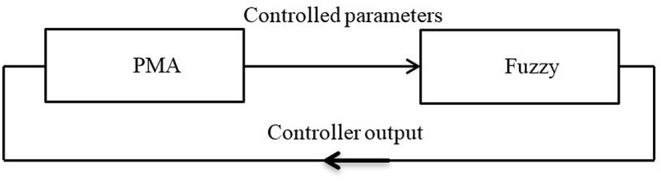
The fuzzy logic block diagram controller system.

The fuzzy control based on bang-bang control strategy is used in Leephakpreeda (2011) with a combination of proportional control to adjust the system output around the desired points either for the length of the contraction force. Figure 2 shows a diagram of this control system. In this method, the author used the pulse width modulation (PWM) technique as a variable time on–off controller to adjust the air valve outlets. The model-based statics controller has been utilized in Camarillo et al. (2009) for a 5-DoF-*per-sec*tion model by formulating IK. The most frequently used of IK-based static controls uses the constant curvature (CC) approximation (Hannan and Walker, 2003).

On the other hand, model-free approaches for control of soft robots are quite a novel field and provide a wide range of possibilities. The early utilization of this method has been proposed in Giorelli et al. (2013) and for a 2- and a 3-DoF cable-driven soft manipulator (Giorelli et al., 2015a,b). The main idea for this controller system is applying a closed-loop control system with an effective sensory feedback system.

## Parallel Neural Network Proportional Controller

**Figure 3:**
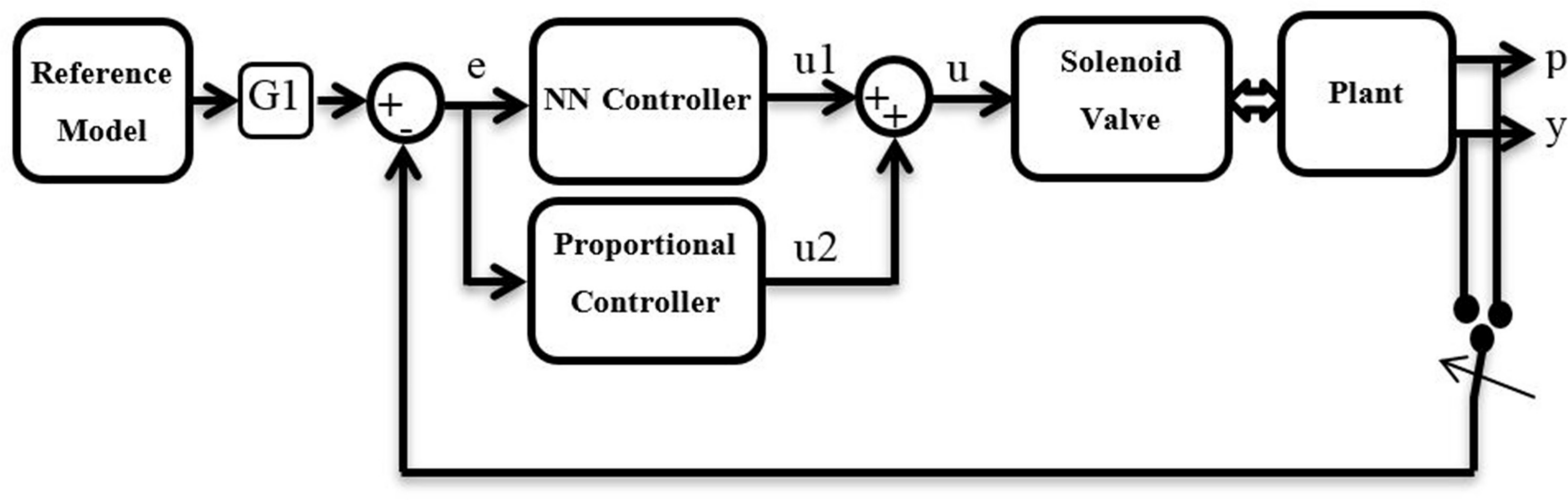
The schematics of the suggested controller.

The parallel neural network proportional (PNNP) controller is suggested in this section. The NARMA-L2 neural network control system has been utilized. The structure of nine neurons has been chosen in a single hidden layer, three delayed controlled signal outputs, and two delayed plant outputs. The NN has been trained by trainlm for 100 Epochs. The mean square error (MSE) for the training, testing, and validating data is about 10^−7^. The NN controller system provides good performance; nonetheless, the PMA system is too slow, and it needs a fast controller to track its behavior. To enhance the speed of the controller system, a proportional controller has been used in parallel to the NN controller. While the NN controller provides high precision, the P controller offers a high-speed response. As a result, the structure of the PNNP controller provides efficient performances in terms of precision and speed. The structure of the controller is shown in Figure 3.

The reference model states the required target such as length, position, bending angle, and so on. Moreover, since the air pressure in single or multiple PMA defines the system performances, G1 can either be the inverse kinematics of the plant and, in this case, the proposed controller will adjust the pressure *p*, or be equal to 1 and, in this case, the controller system will track the error in the output *y*.

The error *e* can be defined as follows:

(4) $$\begin{array}{l} e=p_{r}-p\;,if\;G1\;is\;IK \end{array}$$

or

(5) $$\begin{array}{l} e=y_{r}-y\;,if\;G1\;is\;1 \end{array}$$

*p*_*r*_ and *y*_*r*_ are the reference (setpoint) for the pressure and the system's output, respectively.

The controller outputs *u*1 and *u*2 represent the duty cycle of the PWM signal for the NN controller and P controller, respectively, where

(6) $$\begin{array}{l} u=u1+u2 \end{array}$$

The NARMA-L2 NN controller output *u*1 can be defined as

(7) $$\begin{array}{l} u1\left(k\right)=\frac{y_{r}\left(k+1\right)-f\left[y_{n}\left(k\right),\;{u1}_{m}\left(k-1\right)\right]}{g\left[y_{n}\left(k\right),\;{u1}_{m}\left(k-1\right)\right]} \end{array}$$

where *f*() and *g*() are approximated using neural networks, and

(8) $$\begin{array}{l} y_{n}\left(k\right)={\left[y\left(k\right),\;\ldots ,\;y\left(k-n+1\right)\right]}^{T} \end{array}$$

(9) $$\begin{array}{l} {u1}_{m}\left(k-1\right)={\left[u1\left(k-1\right),\;u1\left(k-2\right),\;\ldots ,\;u1\left(k-m\right)\right]}^{T} \end{array}$$

where *n* and *m* are equal to 2 and 3, respectively, according to the proposed controller structure.

While the proportional controller output has been defined as

(10) $$u2\left(k\right)=k_{p\;}\frac{\left(y_{r}\left(k+1\right)-y(k+1))(u_{\max}\right)}{x}$$

The PWM signal controls the airflow for the valve output in fill and vent directions. Therefore, two PNNP controllers are required: one to control the airflow in the fill direction and the other controls the venting process.

Depending on the error, the proposed controller activates either the filling controller (positive error) or the venting controller (negative error). On the other hand, two possible methods are used to train the NN. The first method is using an approximate function between the output and the duty cycle as in (11):

(11) $$\begin{array}{l} y^{*}=y_{0}+\frac{x\;u^{*}}{98} \end{array}$$

In the case of the pressure controller of the single or multiple actuators, y^*^ and *y*_0_ represent the air pressure in the actuator *p* and the initial pressure in the actuator, respectively, *x* is the maximum applied pressure *p*_*max*_ (*p*_*max*_ is subject to the actuator size and material), and u^*^ is the training duty cycle of the NN controller.

In most cases,

(12) $$\begin{array}{l} p_{\max}=500\;kPa \end{array}$$

(13) $$\begin{array}{l} 0\leq p\leq p_{\max} \end{array}$$

(14) $$\begin{array}{l} x=p_{\max} \end{array}$$

(15) $$\begin{array}{l} 0\leq u^{*}\leq 100 \end{array}$$

In order to prevent a continuously applied voltage (100% duty cycle) on the solenoid valve, we chose 98% as the maximum operating duty cycle. Formula (11) provides an acceptable linear performance of the PMA pressure at variable duty cycles.

Alternatively, the actual relationship between the output and the duty cycle can be found experimentally as follows.

**Figure 4:**
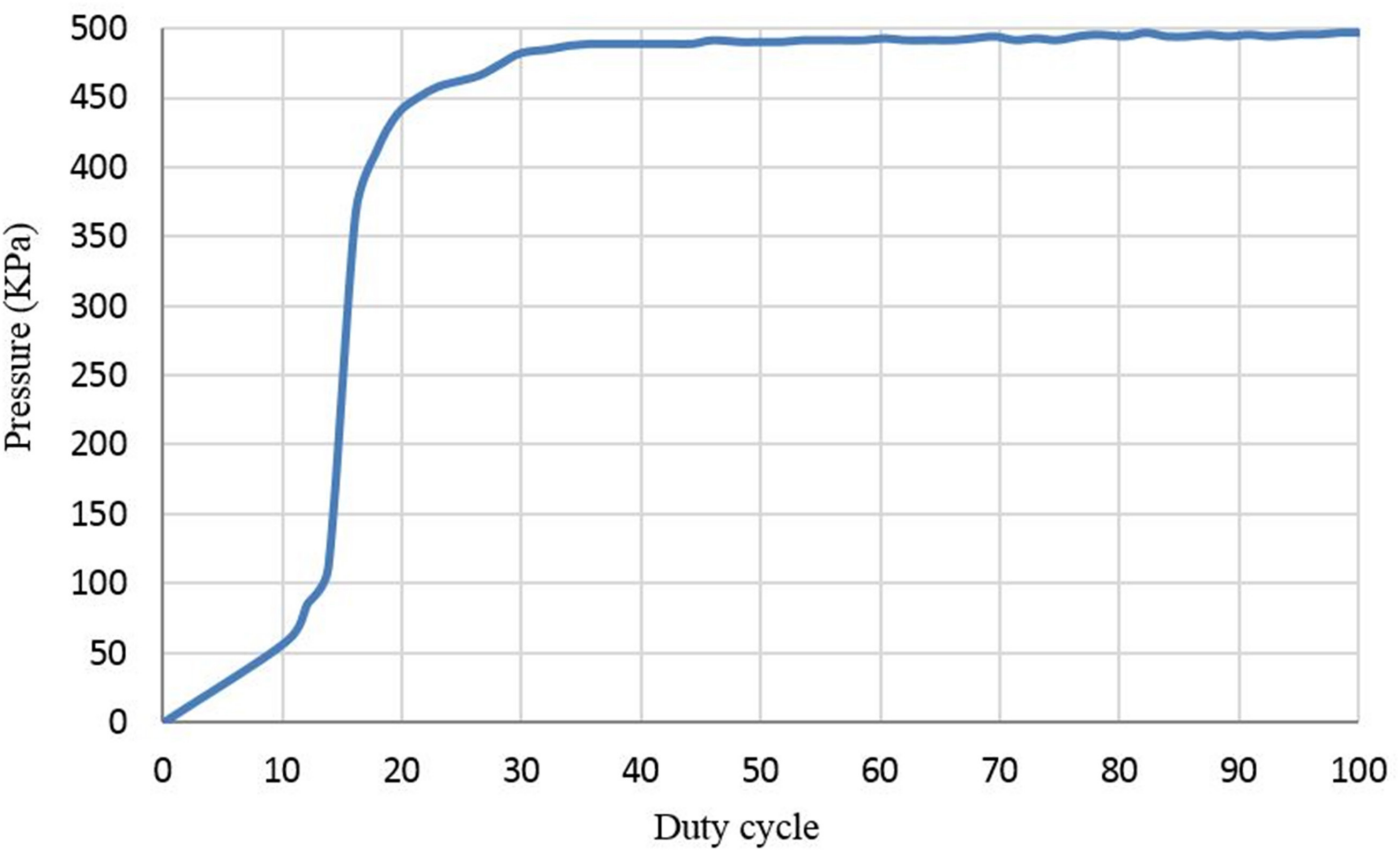
The relation between air pressure and duty cycle.

A contraction actuator of 30 cm in length and 1.7 cm in diameter is chosen. A source of 600 kPa is used to apply air pressure to this actuator via a solenoid valve by different duty cycles ranging from 0 to 100% within 1 s. Firstly, a 10% duty cycle is selected, the air pressure is measured by a pressure sensor, and then the venting process is activated. This process is repeated for 20, 30…, and 100%, respectively. The result of this experiment is shown in Figure 4. The trained line to these data is utilized for training the NN.

Formula (11) is used due to the similarity in the performances of the NN and to decrease the complexity of the control system. Moreover, the PNNP controller is tracking the desired behavior online; therefore, the controller is adjusting the duty cycle to minimize the error.

### Length Control of Single Extensor PMA

In order to validate the proposed controller, a 30-cm extensor actuator is chosen. To measure the change in length of the extensor PMA, an ultrasound HC-SR04 sensor is fixed to the end of the air muscle. According to (11), y^*^ is the length of the actuator *L, y*_0_ is the initial length *L*_0_ of the extensor PMA (30 cm), and *x* is the maximum extension ratio (50% of *L*_0_).

**Figure 5:**
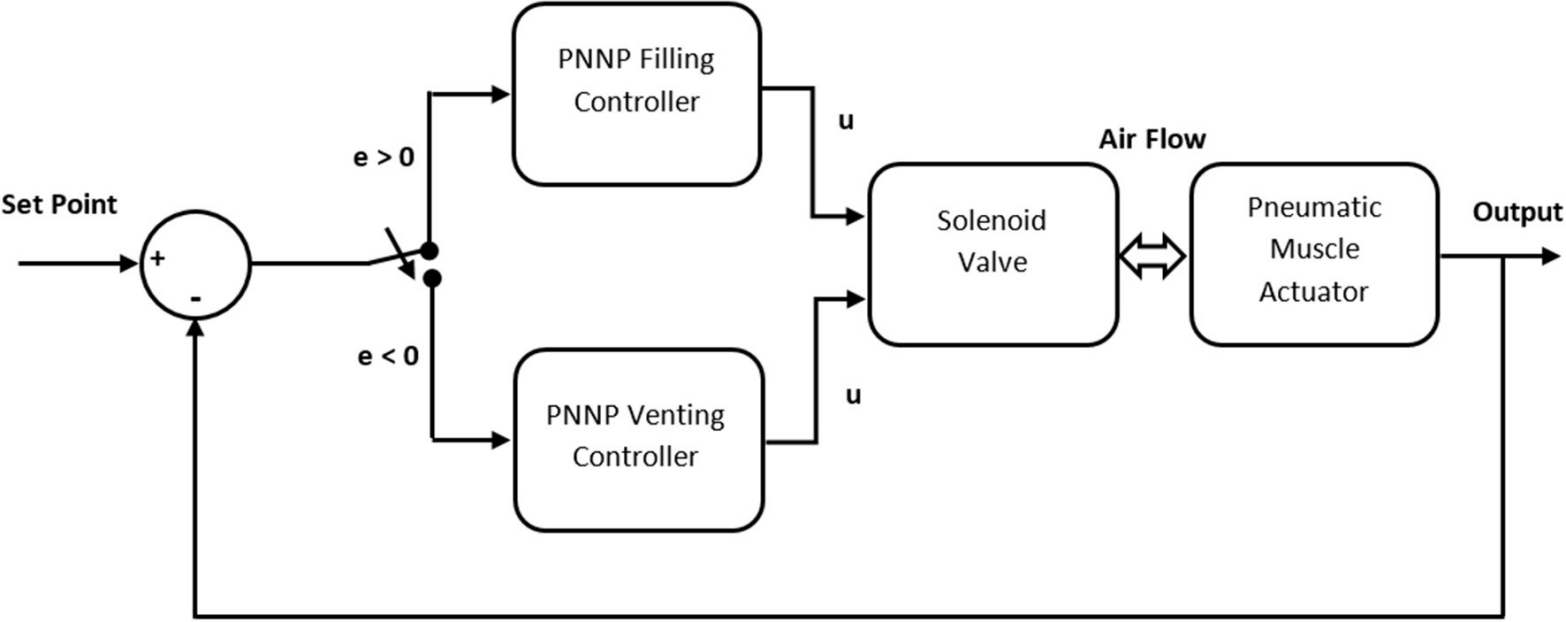
The structure of the proposed controller for single PMA.

The PNNP controller sends the controlled input *u* to the (3/3 Matrix MK 754.8E1D2XX) solenoid valve via Arduino Mega 2560. The Arduino acts as an interface between the PC and the valve-actuator system. It is reading the pressure and the distance from the pressure sensor and the ultrasound sensor and sending them to the Matlab *via* a USB port. Then, The PNNP controller adjusts the duty cycles for both the filling and the venting and sends them back to the valve as follows:

While the actuator air pressure is low and the PMA length is less than the required, the error will be positive and that activates the filling PNNP controller branch (see Figure 5) to actuate the extensor muscle and increase its length. The controlled duty cycle *u* decreases gradually according to the feedback error until the error becomes zero; at this point, the filling PNNP controller is being inactivated. Due to the hysteresis behavior of the PMA, the length of the actuator will be slightly increasing, which leads to a negative error. The venting PNNP controller responds to the increment of the actuator length by decreasing the amount of pressure. The expected maximum controlled duty cycle is low because of the small error value. As a result, the length of the actuator decreases until the error reaches zero. This process of filling and venting might be repeated several times according to the sign of the feedback error. The operation at low frequencies decreases the number of the filling and venting controlling process repetitions because the whole controlling process speed operates close to the pneumatic system behaviors.

Attaching a load or increasing its value causes increasing in the actuator length. To keep the length as required by the reference model, the venting controller operates to reduce the length, and, of course, the filling will be activated if overcontraction occurs.

**Figure 6:**
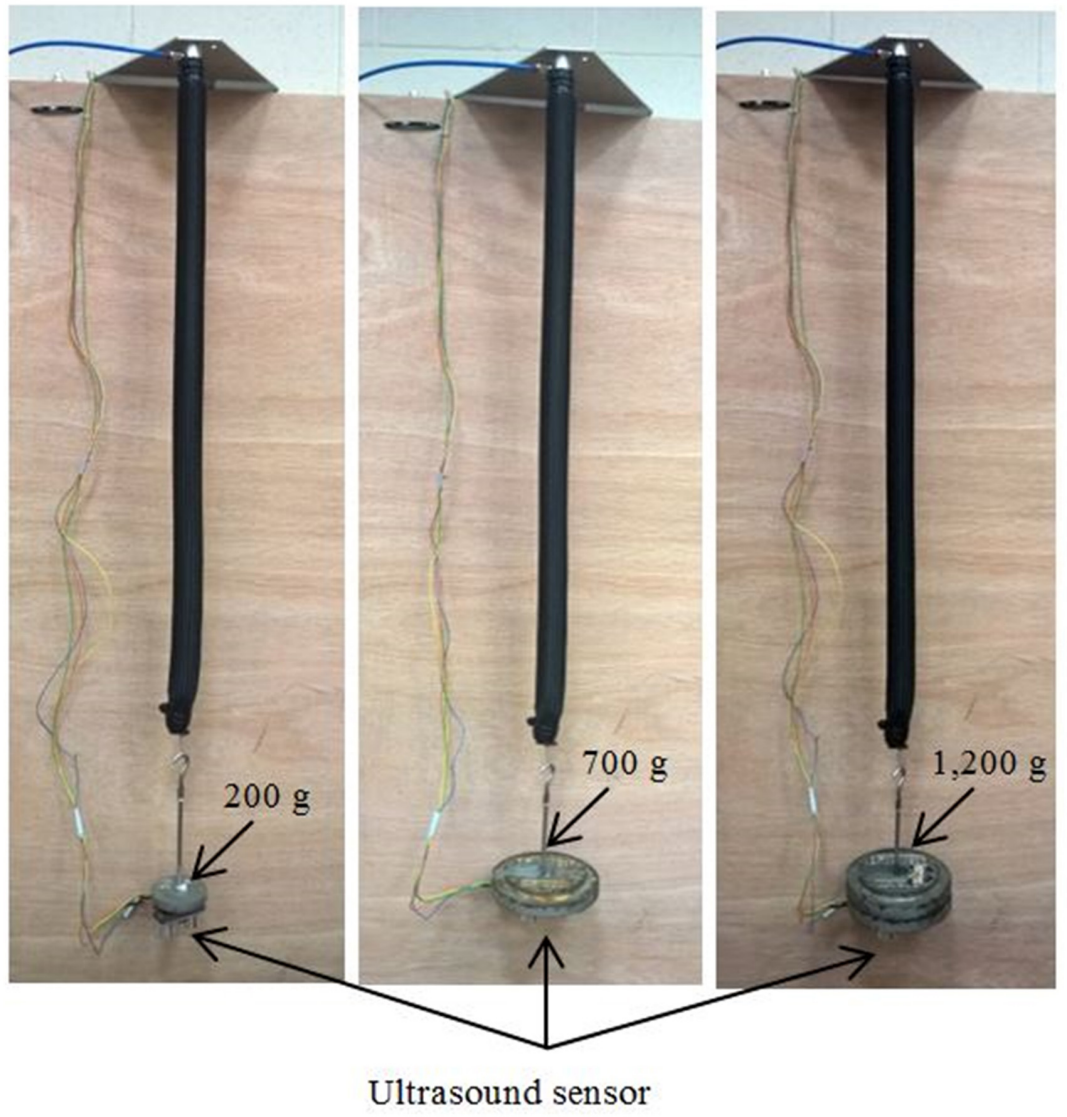
The photograph of the extensor actuator at various loads and the position of the attached ultrasound sensor.

**Figure 7:**
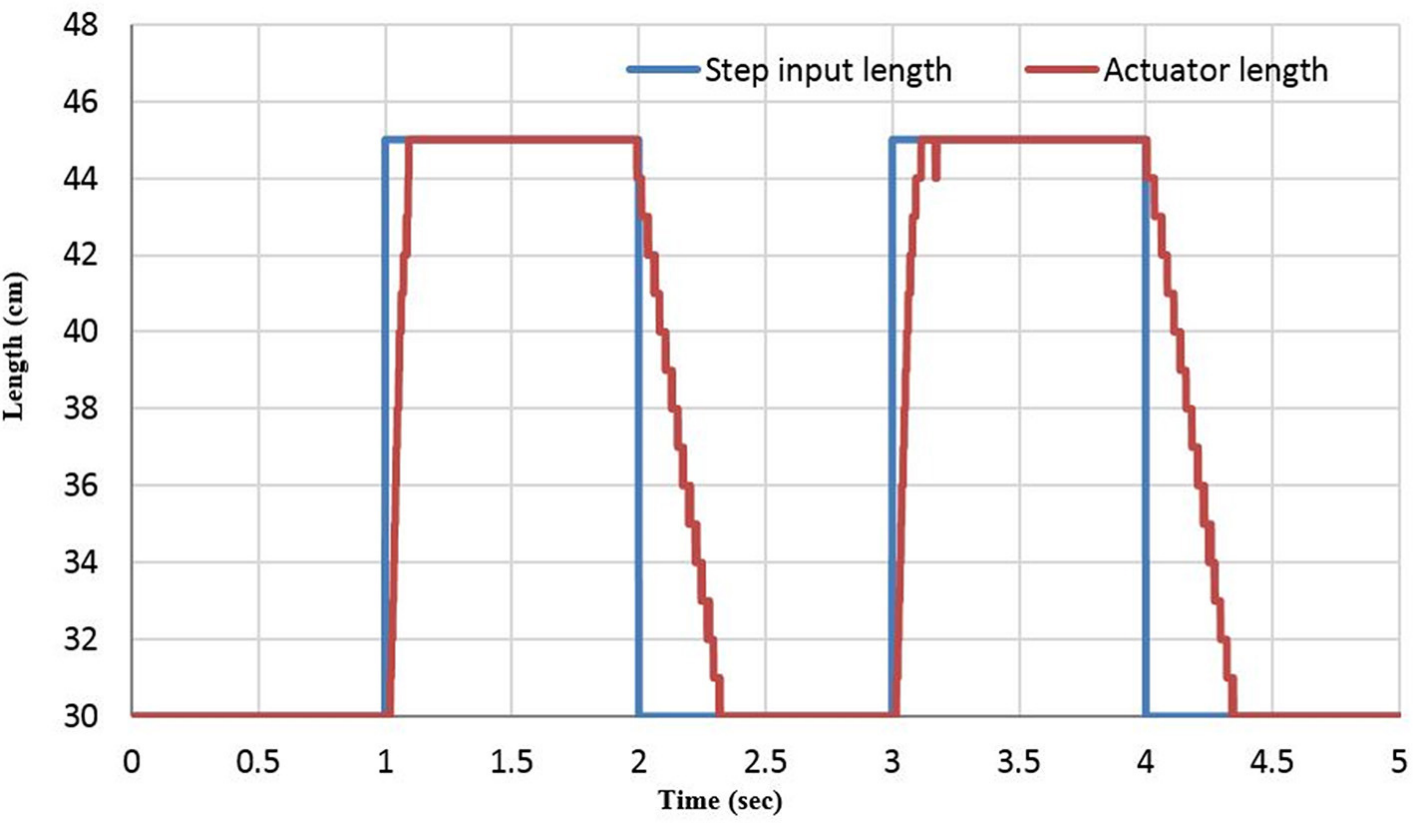
The unit step response of the length controller system at 0.5 Hz.

The length of the actuator is controlled under three different load values. At each time, a square wave between 30 and 45 cm is applied as a reference at 0.5 Hz. The extensor actuator and the control performance are illustrated in Figures 6, 7, respectively, for 200 g.

Figure 7 shows that the venting time takes longer than the filling time due to the hysteresis of the actuator material and the air pressure difference between the environments.

### Bending Angle Control of Single SBCA

The presented SBCA in Al-Ibadi et al. (2020) has been used in this section to control its bending angle. The specifications of the bending actuator are listed in Table 1.

**Table 1:** The dimensions and material specifications of the SBCA.

***L*_0_ (m)**	**Rubber thickness (m)**	**Braided thickness (m)**	**Inner diameter (m)**	**Rubber stiffness(N/m)**	**Rod length (m)**	**Rod thickness (m)**	**Rod width (m)**
0.3	1.1 × 10^−3^	0.5 × 10^−3^	12 × 10^−3^	363.33	0.3	0.002	0.006

**Figure 8:**
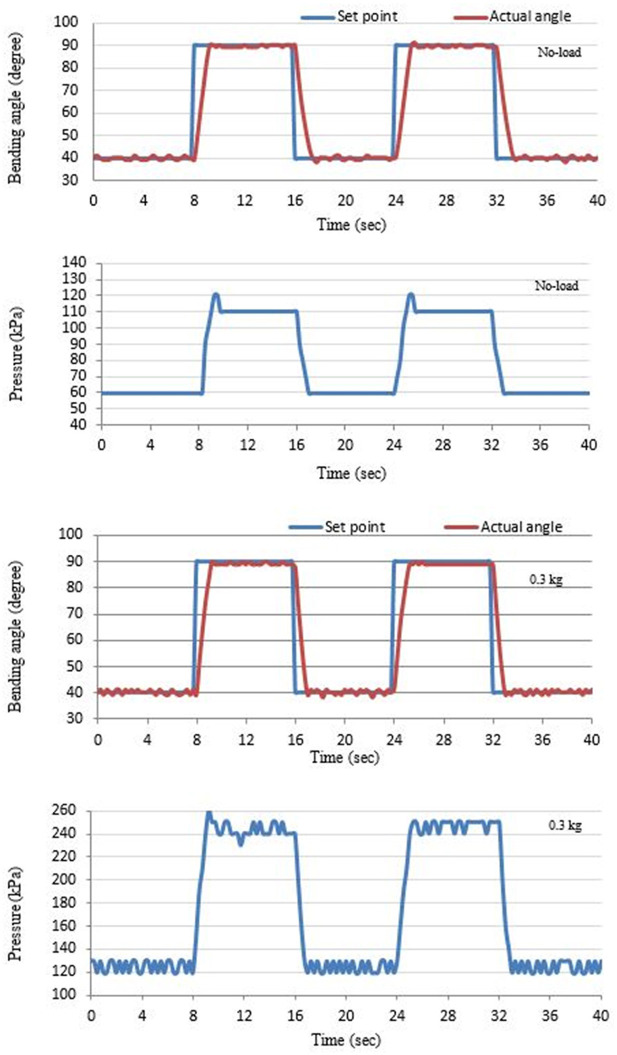
The bending angle and the applied pressure of the SBCA at no load and 0.3 kg.

A similar controller has been used to control the bending angle at different load values, but, in this case, the initial bending angle is zero, and the maximum bending angle for the chosen specification in Table 1 is 135° at 500 kPa. Square wave between 40 and 90 is selected as a reference model for tracking the bending angle of the bending actuator between 40 and 90°. Figure 8 illustrates the controller response at no load and 0.3 kg. Pressure and MPU sensors are used to record the actuator air pressure and the bending angle, respectively. The MPU is mounted at the free end of the SBCA. A similar procedure to the extensor actuator can be shown here; the positive 40° feedback error triggers the filling PNNP to apply air pressure to the SBCA. The actuator bending angle increases to 40°. The zero-feedback error isolates the filling controller to avoid increasing the bending angle. Again, because of the non-linear behaviors of the PMA, the bending angle might increase by some degrees. This leads to the activation of the venting controller until the error reaches zero again. Similar processes are applied to the second value of the reference signal (90°). For the second cycle of the reference square signal, the venting controller operates first, and the filling PNNP responds to the decreasing bending angle. Furthermore, increasing the load value at any moment leads to the reduction of the bending angle, and more air pressure is required to reduce the positive error by the filling controller.

Figure 8 shows that the controller system applied more air pressure when the load is increased to reach the required bending angle.

### Human–Robot (H–R) Interaction (HRI)

**Figure 9:**
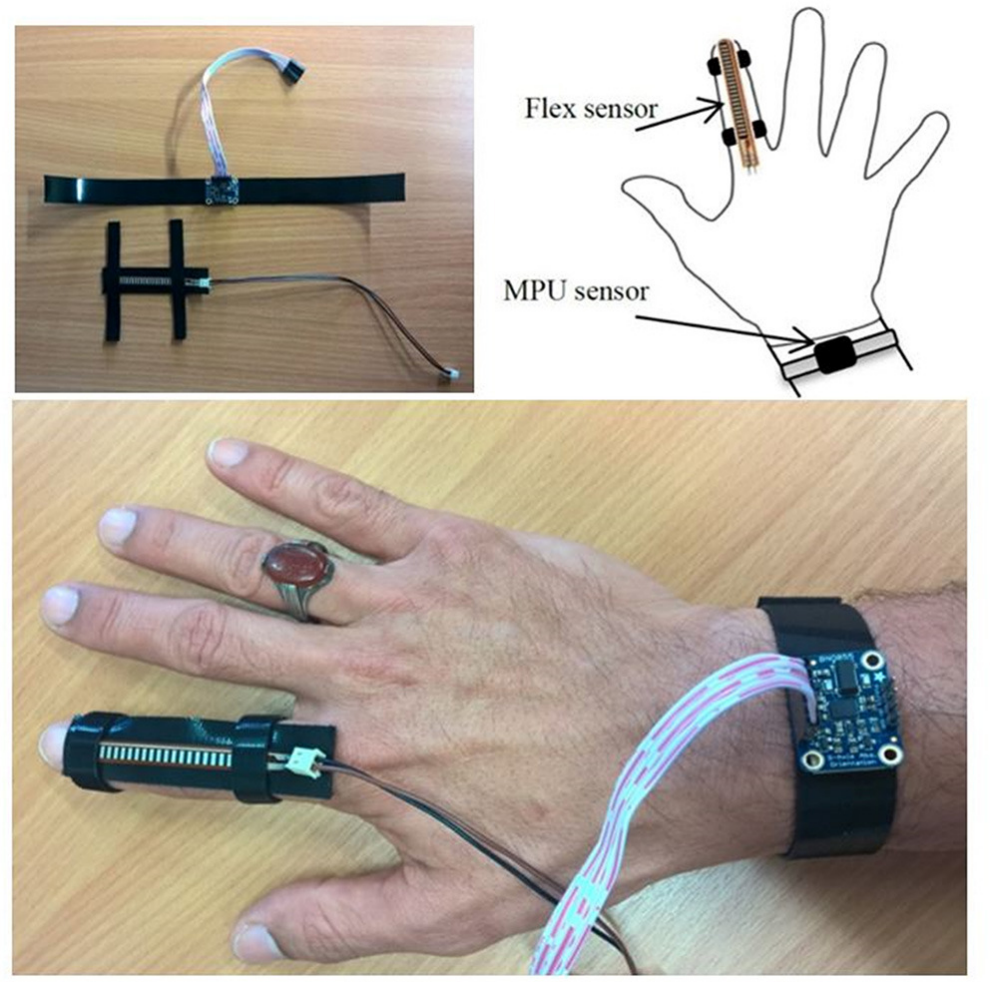
The wearable sensors to control the bending angle and grasping force.

Unsafe workspaces for individuals force them to work from a split site. In this section, a unidirectional continuum arm and a four-finger gripper are used to work in a workspace considered to be unsafe for a human being. The MPU and the pressure sensors are used to measure the bending angle of the continuum arm and the air pressure in the finger gripper, respectively. On the other hand, another MPU sensor and a flex sensor are worn by a human hand, as shown in Figure 9.

The wearable MPU sensor is used to send the set bending angle to the PNNP controller to adjust the bending angle of the continuum arm, and the flex sensor controls the grasping force of the four-finger gripper by converting the resistance to pressure by mapping its data at different bending steps for the index finger. The control system controls the air pressure in the fingers to control the grasping process.

**Figure 10:**
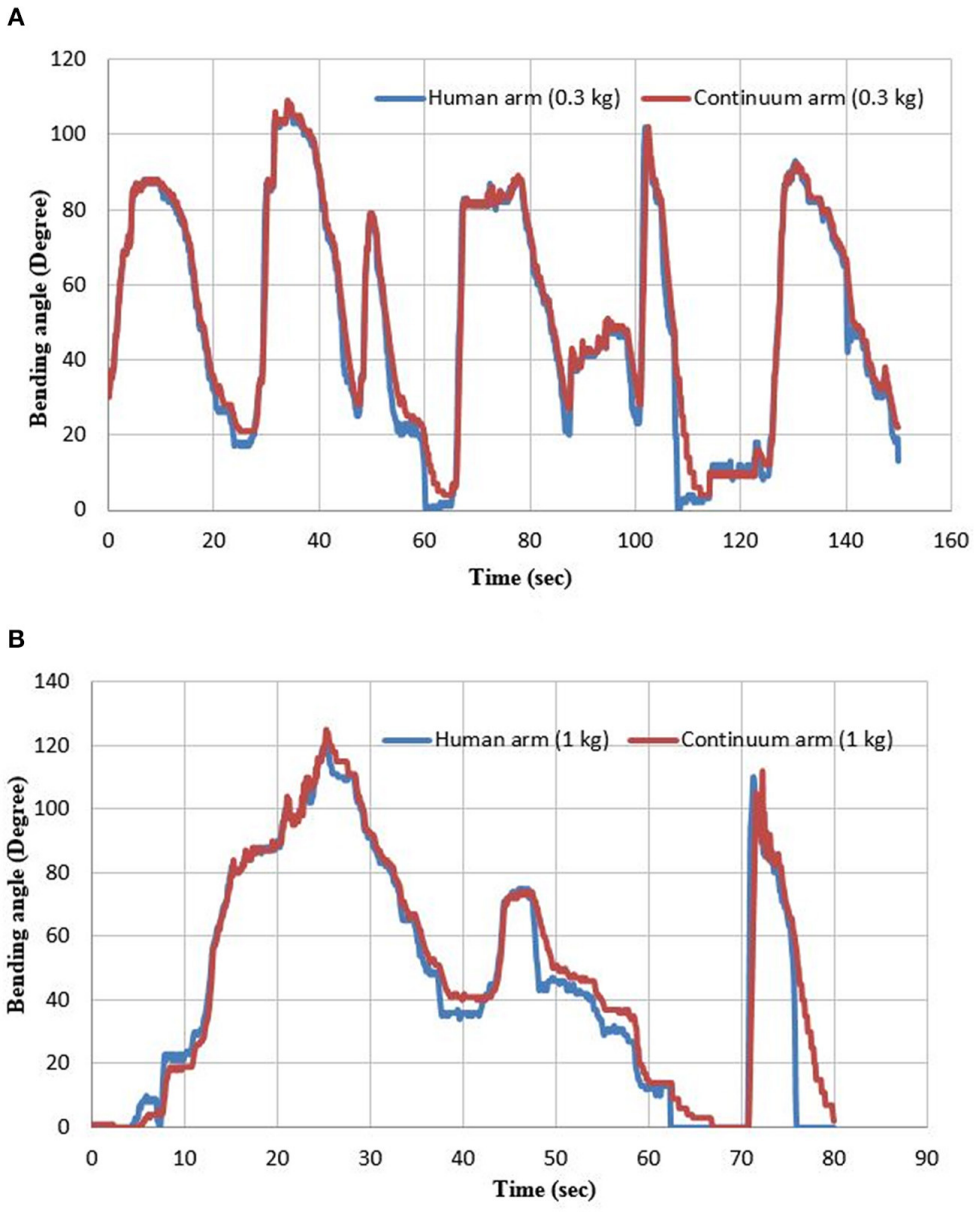
The bending angle for both the human hand and the continuum arm at **(A)** 0.3 and **(B)** 1 kg.

In this process, the human sends a variable reference bending and grasping force to the controller through the Arduino Mega 2560, and the controller adjusts both of them on the continuum arm. Figure 10 shows the bending angle for both the human hand and the continuum arm at two different loads.

Since the application consists of two pneumatic systems, four PNNP controllers are required, two filling controllers and two venting controllers. Either the filling PNNP or the venting PNNP controllers are being activated for the gripper to adjust the grasping force as required by the reference value, which is sent by the human index finger. Simultaneously, the bending angle of the human arm is sending to another group of PNNP controllers to adjust the bending angle of the continuum arm.

Figure 10 illustrates the efficiency of the PNNP controller, which provides precise tracking for the bending angle of the human arm. As previously mentioned, the tracking error for the filling process is less than the error of the venting process due to the variations between the air pressure in the two different environments.

The comparison with the literature shows that the performance error is very low for the three presented applications. While it is seen obviously at numerous previous researches, such as the performance error for the ankle rehabilitation robot in Meng et al. (2017), the possible cause for that is using the PID controller to control the high non-linear system (the PMA).

## Comparison With Previous Controller Approaches

The PNNP controller shows efficient performances when it is applied to soft pneumatic systems. In this section, the PNNP is compared with several other controller approaches from the literature to show the advantages of the proposed controller system. Table 2 lists the main characteristics of numerous controllers.

**Table 2:** The dimensions and material specifications of the SBCA.

**Research title**	**Type of controller**	**Linearity**	**Performance**
The Proposed Controller (current article)	Parallel neural network proportional (PNNP)	Non-linear	High response (settling time = 0.15 s at 0.5 Hz), accurate, suitable for single and multiple actuator systems, provides local controlling for every single actuator
Andrikopoulos et al. (2011)	PID and on–off	Linear	Low response (settling time = 16 s), tested for a climbing robot of four PMAs
Chan et al. (2020)	Cascaded PID	Linear	Low response (settling time = 2 s) and tested for single PMA
Shen et al. (2015)	PID	Linear	Moderate response, tested to control a robot leg of two PMAs
Meng et al. (2017)	Iterative feedback tuning control (IF-PID)	Linear	Moderate response (high settling time, the error does not reach zero), tested for four single actuators for ankle rehabilitation system
Chiang and Chen (2017)	Neural network fuzzy sliding mode controller	Non-linear	High performance at frequencies ≤ 0.05 Hz and low performance at 1 Hz
Chan et al. (2003)	Fuzzy PD+I	Non-linear	Low response (1–2 s), tested for single PMA
Andrikopoulos et al. (2014)	Advanced non-linear PID	Non-linear	Moderate response (settling time = 0.5 s at 0.25 Hz), tested for single PMA

Table 2 shows that the performance of the PNNP controller system in terms of speed response, accuracy, and applications (complexity of the pneumatic system) is higher than other chosen researches.

## Conclusion

The high non-linear behaviors of the pneumatic muscle actuator require fast response and high accuracy control systems. In this article, a parallel structure of the neural network controller and the proportional controller is presented to control single extensor PMA and single SBCA, respectively, at different load values. For further validation of the PNNP controller, an interaction between a human and a bidirectional continuum arm has been designed, and the controller system shows a valuable tracking to the human hand. The results illustrated the efficiency of using the parallel structure to increase precision and decrease the tracking time.

The results show that the venting time is more than the filling time due to the non-linear behavior of the PMA such as hysteresis and the air pressure difference inside and outside the actuator. Furthermore, increasing the load for the presented pneumatic systems does not have any effect on the resulting performances. Nonetheless, the PNNP decreases the required air pressure for the extensor PMA at a higher load to decrease the extension ratio, while the proposed controller increases the applied air pressure for the single SBCA and the unidirectional continuum arm to raise the bending angle. The actuators are tested at pressure up to 600 kPa, but the maximum air pressure has been set at 500 kPa for safe working.

## Data Availability Statement

All datasets presented in this study are included in the article/Supplementary Material.

## Ethics Statement

Written informed consent was obtained from the individual(s) for the publication of any potentially identifiable images or data included in this article.

## Author Contributions

AA-I designed, performed the experiments, and wrote the paper. AA-I and SD analyzed the data. SN-M edited the paper. All authors have read and agreed to the published version of the manuscript.

## Conflict of Interest

The authors declare that the research was conducted in the absence of any commercial or financial relationships that could be construed as a potential conflict of interest.
